# Carbon Dioxide Potentiates Flucytosine Susceptibility in Cryptococcus neoformans

**DOI:** 10.1128/spectrum.04783-22

**Published:** 2023-01-31

**Authors:** Andrew J. Jezewski, Laura C. Ristow, Damian J. Krysan

**Affiliations:** a Department of Pediatrics, Carver College of Medicine, University of Iowa, Iowa City, Iowa, USA; b Department of Microbiology and Immunology, Carver College of Medicine, University of Iowa, Iowa City, Iowa, USA; Stony Brook University

**Keywords:** *Cryptococcus neoformans*, antifungal drug, flucytosine

## Abstract

Cryptococcal meningoencephalitis remains a global health threat with limited treatment options. Currently, the most effective treatment regimens are based on a combination therapy of flucytosine with either amphotericin B or fluconazole. Slow but steady progress is being made toward universal access to flucytosine-based therapies. The broadening access to flucytosine combination therapies will be accompanied by the need for microbiological methods that reliably determine strain susceptibility. This is especially true considering that flucytosine susceptibility can vary widely across clinical isolates. Identifying culture conditions that best represent the host environment are likely optimal and may even be required for accurately determining *in vivo* flucytosine susceptibility. Here, we report that culture conditions incorporating host-like concentrations of carbon dioxide (CO_2_) potentiated flucytosine susceptibilities across clinical isolates (10 of 11) that exhibited a range of MIC values under ambient growth conditions (2 to 8 μg/mL) by standard Clinical and Laboratory Standards Institute susceptibility testing. CO_2_ induced a dose-dependent increase in flucytosine susceptibility between 2- and 8-fold over standard conditions. The CO_2_-dependent increase in flucytosine susceptibility did not correspond to an increase in fluorouracil susceptibility, indicating a central role for flucytosine uptake through the cytosine permease in the presence of host-like CO_2_ concentrations. Indeed, the expression of the cytosine permease gene (*FCY2*) was induced 18- to 60-fold in the mouse lung environment. Therefore, the activity of flucytosine is likely to be very dependent upon host environment and may not be well represented by standard *in vitro* susceptibility testing.

**IMPORTANCE** Cryptococcus neoformans causes life-threatening infections of the brain. The most effective treatment regimens are based on flucytosine-based combination therapy, which has led to increasingly successful broadening of access to flucytosine globally. Wider use of flucytosine-based therapies for cryptococcal infections will require the ability to reliably determine clinical isolate susceptibilities. We showed that host-like carbon dioxide stress affected flucytosine susceptibility, and this likely occurred through flucytosine uptake. We further showed that the gene encoding the permease, *FCY2*, and that is responsible for flucytosine uptake was strongly induced during cryptococcal infection. Our data provide insights into the distinctions between the activity of flucytosine in the host environment and during *in vitro* susceptibility testing.

## OBSERVATION

Cryptococcus neoformans is a frequent cause of life-threatening fungal infections that primarily occur in immunocompromised patient populations (1). Although cryptococcosis most commonly affects people living with HIV, its prevalence is also a concern among patients receiving immune-suppressive therapies to manage autoimmune disease or solid organ transplantation. Amphotericin B (a polyene) is the long-standing gold standard treatment for cryptococcosis. Unfortunately, amphotericin B often requires hospitalization and is therefore a less amenable treatment option in resource-limited regions of endemicity. This has left the more tolerated yet far less effective fluconazole (an azole) as a frontline therapy for some regions of the world. Beyond polyenes and azoles, no other class of antifungals serves as a viable anticryptococcal monotherapy. A third antifungal drug, flucytosine (5-FC), improves treatment outcomes when combined with either amphotericin B or fluconazole. Clinical trials investigating either amphotericin B or fluconazole in combination with flucytosine showed a 1.5- to 3-fold increase in survival over the respective monotherapies (2, 3). Moreover, flucytosine has been reported to suppress heteroresistance to fluconazole (4). The accumulating clinical data strongly support the use of flucytosine as an adjunctive therapy. Fortunately, flucytosine is becoming more accessible and more widely adopted. Understanding the mechanisms that direct flucytosine as an effective adjuvant will be important for identifying the underlying factors that determine treatment outcome.

The apparent *in vivo* synergistic effects offered by flucytosine combination therapy have been somewhat perplexing compared to the *in vitro* data. Clinical isolates show a lower rate of synergism *in vitro* compared to the positive *in vivo* outcomes observed in combination with amphotericin B or fluconazole (5, 6). This disconnect between *in vitro* and *in vivo* flucytosine efficacy has also been observed in Aspergillus fumigatus and less so for Candida albicans (7–9). Discrepancies between *in vitro* testing conditions and the *in vivo* infection environment may contribute to this discordance. As part of a project to identify *in vitro* Cryptococcus susceptibility testing conditions for flucytosine that better correlated with *in vivo* outcomes, Viviani and colleagues found that yeast nitrogen base rather than the standard RPMI medium and pH 5.4 rather than pH 7.0 were conditions that best correlated with *in vivo* flucytosine efficacy (10). Separately, Verweij and colleagues observed that testing flucytosine susceptibility against Aspergillus at pH 5.0 rather than pH 7.0 was a far better predictor of *in vivo* efficacy (9). Later, the pH dependence of flucytosine activity against Aspergillus fumigatus was determined to result from a pH-dependent regulation of the cytosine permease, encoded by *FCYB*, that mediates flucytosine uptake (11). We present data that this mechanism is also present for C. neoformans but that an alternative stress derived from host carbon dioxide (CO_2_) concentrations may contribute to enhanced flucytosine susceptibility *in vivo*.

**FIG 1:**
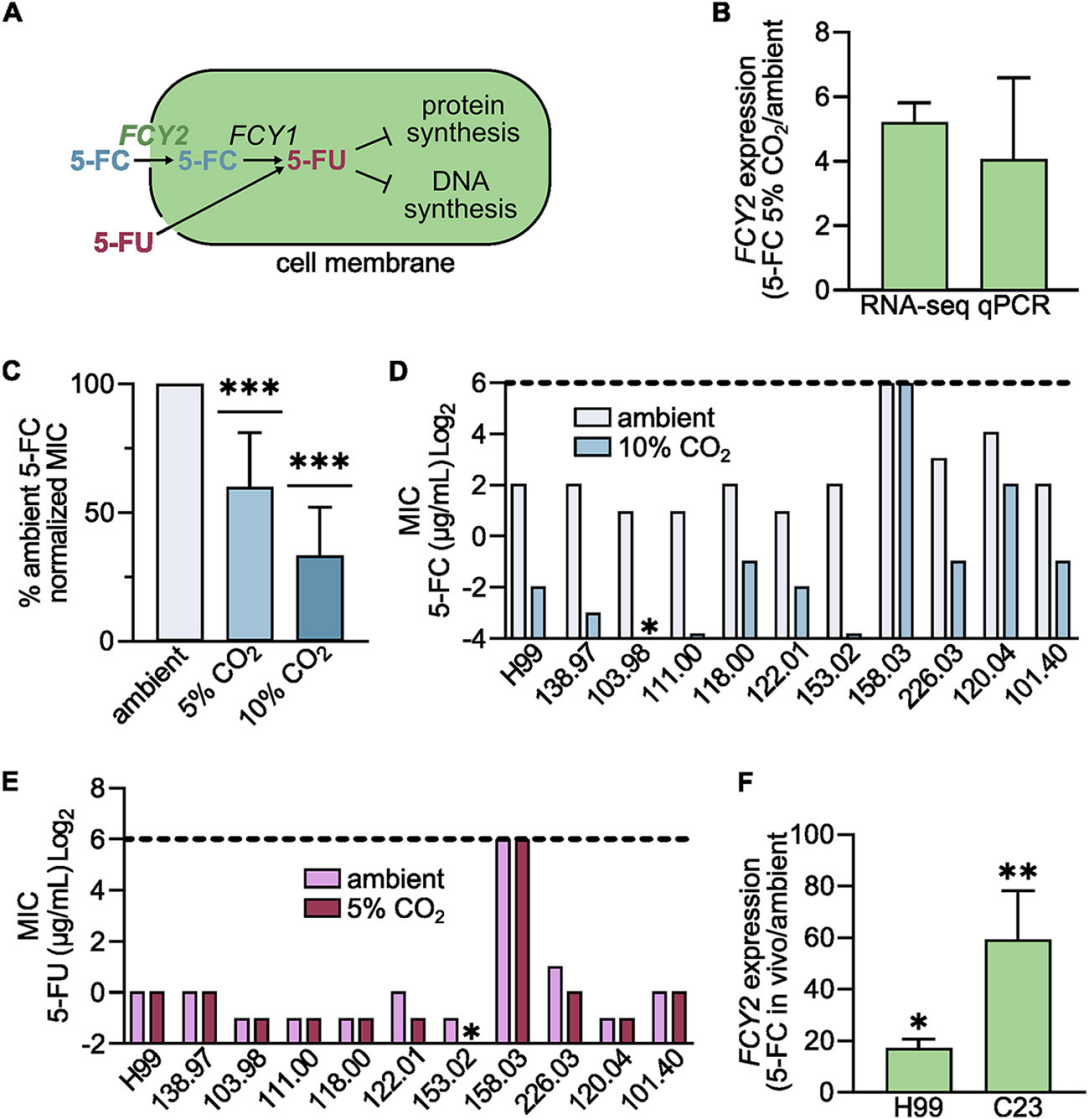
Carbon dioxide potentiates 5-FC susceptibility through *FCY2* in C. neoformans. (A) Schematic of 5-FC uptake through the cytosine permease (*FCY2*) and processing by the cytosine deaminase via *FCY1* to 5-FU. (B) Relative expression of *FCY2* in RPMI-MOPS (pH 7.0) with RPKM reads in 5% CO_2_ relative to that under ambient air conditions for RNA-seq and threshold cycle (ΔΔ*C_t_*) of *FCY2* normalized those of *ACT1* for qPCR. (C) MICs determined via CLSI standards modified with 5% or 10% CO_2_ and normalized to ambient air conditions for 5-FC. Inferential statistics were performed using GraphPad Prism and a one-sample *t* test against ambient conditions. ***, *P* < 0.001. (D) MICs determined via CLSI standards with ambient air or 10% CO_2_ at 30°C; asterisk denotes no growth under tested conditions. (E) MICs determined via CLSI standard methods under ambient air for 5% CO_2_ for 5-FU; asterisk denotes no growth under tested conditions. (F) Relative expression of *FCY2* on day 4 of intranasal pulmonary murine infection, normalized to expression of *ACT1* and compared to *in vitro* ambient RPMI-MOPS (pH 7.0) growth conditions via qPCR. Inferential statistics were performed using GraphPad Prism and a one-sample *t* test against ambient *in vitro* conditions. *, *P* < 0.05; **, *P* < 0.01. All clinical strains were C. neoformans var. *grubii* and were generously provided by John Perfect at Duke University. Numbers denote strain identifiers.

For environmental fungi such as C. neoformans and A. fumigatus to initiate infection, they must first adapt to the lung environment. We have recently shown that an elevated CO_2_ concentration (~5%) in the lung relative to ambient air (0.001%) is an independent stress, separate from pH, that C. neoformans must overcome to establish infection in mammalian hosts (12). Additionally, we reported that host-like concentrations of CO_2_ (5%) potentiated the antifungal activity of azoles, including fluconazole, toward C. neoformans. In contrast, CO_2_ does not affect susceptibility to polyenes such as amphotericin B (12). To understand the underlying mechanisms contributing to these observations, our ongoing work (to be reported elsewhere) includes RNA sequencing (RNA-seq) analysis to assess the transcriptional response of the C. neoformans reference strain H99 to CO_2_ stress in RPMI-morpholinepropanesulfonic acid (MOPS) medium at pH 7.0, with or without 5% CO_2_. This work uncovered the fact that expression of the cytosine permease gene *FCY2* was elevated in cultures incubated at 5% CO_2_ relative to those under ambient CO_2_ conditions (fold increase of 5.2 ± 0.6 under 5% CO_2_ versus ambient conditions, mean ± standard deviation [SD]; *n* = 3) (Fig. 1B). We repeated this experiment and confirmed this observation through quantitative PCR (qPCR) analysis (4.1 ± 2.5 increase under CO_2_ versus ambient; *n* = 3) (Fig. 1B). We were curious if this CO_2_-mediated phenomenon could potentiate flucytosine susceptibility. Multiple clinical isolates were tested using the standard CLSI method of antifungal susceptibility testing under either ambient air conditions or under CO_2_ (5%) supplemented air. Across 11 clinical isolates of C. neoformans (generously provided by John Perfect at Duke University), 8 isolates exhibited a 2-fold reduction in the flucytosine MIC, indicating a modest but consistent increase in flucytosine susceptibility in the presence of host CO_2_ concentrations (Fig. 1C). This CO_2_ effect was dose dependent, and 6/11 isolates exhibited a 2- to 8-fold reduction in the MIC under elevated CO_2_ (10%) conditions at 37°C, while the remaining 5 isolates grew too slowly under 10% CO_2_ for MIC determinations (Fig. 1C). We then repeated 5-FC determinations under ambient and 10% CO_2_ with 30°C, and 9/11 isolates exhibited a 2- to 64-fold reduction in MIC, with an average reduction of 15-fold and with 1 isolate unable to grow under 10% CO_2_ (Fig. 1D). The increase in flucytosine susceptibility occurred across strains that exhibited a range of MIC values under ambient growth conditions (2 to 8 μg/mL). The single clinical isolate that did not exhibit increased flucytosine susceptibility under CO_2_ culture conditions had a very high MIC and was likely resistant (64 μg/mL).

5-FC is a synthetic fluorinated analog of cytosine that relies on uptake through the cytosine permease (*FCY2*) (Fig. 1A). Once in the cell, flucytosine is a prodrug that is first converted to 5-fluorouracil (5-FU) and is either incorporated into RNA to inhibit protein synthesis or into DNA to inhibit replication (13). The most common mechanism of flucytosine resistance is loss of Fcy2 function, which prevents flucytosine uptake (13). To assess whether CO_2_ exerts its effect on flucytosine susceptibility through modulation of Fcy2, we characterized the activity of 5-FU against the same clinical isolates in the presence and absence of CO_2_ (5%). Uptake of 5-FU was Fcy2 independent. Therefore, CO_2_ concentrations would have no effect on 5-FU activity if it is acting through its permease function. CO_2_ did not increase the 5-FU susceptibility of the tested strains, consistent with an Fcy2-dependent mechanism for CO_2_-induced flucytosine potentiation (Fig. 1E). This observation is consistent with our RNA-seq data, which showed a >2-fold induction of the cytosine deaminase Fcy1 responsible for converting 5-FC to 5-FU (data not shown). Together, these data suggested that CO_2_ modulates 5-FC susceptibility by modulating cellular uptake rather than processing and incorporation.

Next, we characterized how *in vivo* conditions within the lung affected *FCY2* expression. To assess this, we inoculated A/J mice intranasally with 5 × 10^5^ CFU of either the reference strain H99 or the clinical isolate C23. Lungs of the infected mice were harvested on day 4 postinoculation, and RNA was extracted for qPCR analysis of *FCY2* expression *in vivo*. The H99 (17.7 ± 2.9 increase, *in vivo* versus *in vitro*; *n* = 3) and C23 (60 ± 18.4 increase *in vivo*/*in vitro*; *n* = 5) exhibited higher *FCY2* expression *in vivo* under *in vitro* culture conditions than under ambient conditions (Fig. 1F). This increased *FCY2* expression *in vivo* exceeded the increased expression due to CO_2_ stress alone (Fig. 1B). This suggested that CO_2_ as well as other factors within the *in vivo* environment contribute to elevated expression of *FCY2* in infected lung relative to *in vitro* susceptibility testing conditions.

**FIG 2:**
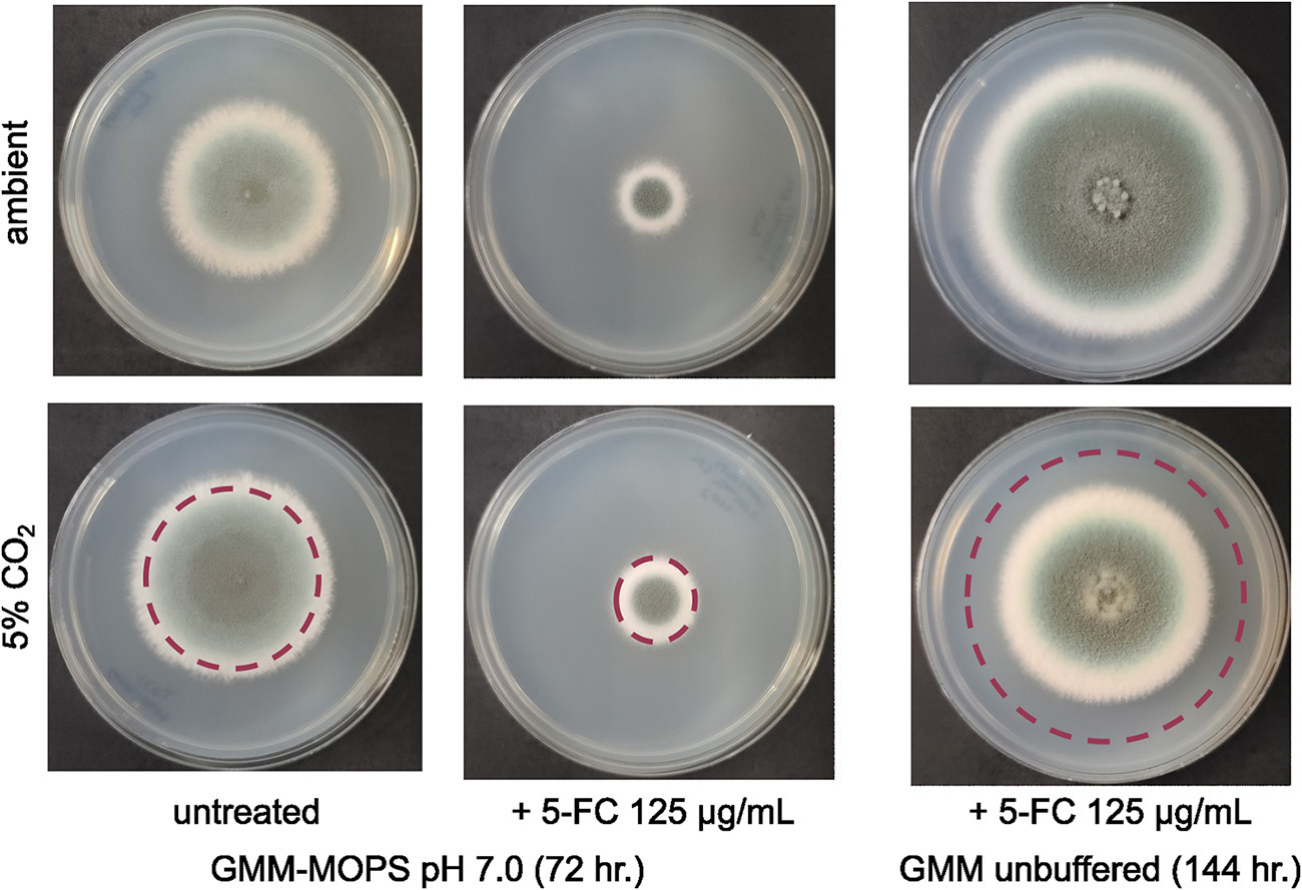
Carbon dioxide does not potentiate 5-FC susceptibility in A. fumigatus. Radial growth assay of 10^3^ conidia of A. fumigatus strain CEA10 on GMM-MOPS buffered at pH 7.0 versus unbuffered GMM plates grown under ambient or 5% CO_2_ supplemented air. Marked circles outline the edge of radial growth for the matched ambient growth conditions.

To further explore the role of host levels of CO_2_ in modulating flucytosine susceptibility, we asked whether CO_2_ rather than pH could also serve as the *in vivo* signal potentiating flucytosine susceptibility against A. fumigatus. We assessed this by using a radial growth assay in glucose minimal medium (GMM) with MOPS buffered at pH 7.0 with 125 μg/mL flucytosine at 37°C under ambient or 5% CO_2_ supplemented air (Fig. 2). No reduction in radial growth was observed under changed CO_2_ conditions, leaving pH-dependent flucytosine potentiation as a the currently supported mechanism. It is possible that exposure to the low-pH environment of the phagolysosome of alveolar macrophages is a driving force behind the pH dependence of flucytosine efficacy. Alternatively, tight control of lung pH has also been shown to be greatly depend on rapid and efficient gas exchange when injured lungs experience a significant drop in pH (14). Perhaps immune infiltration and lung damage associated with disease progression are enough to disrupt the gas exchange and buffering capacity of the lung. Repeating our radial growth assay as before, but without MOPS buffer, revealed 5-FC potentiation with reduced radial growth under 5% CO_2_ relative to plates incubated at ambient CO_2_ concentrations at later time points (144 h) (Fig. 2).

*In vitro* flucytosine susceptibility is highly dependent on the specific culture conditions and does not necessarily correlate well with its activity in animal models or for patient outcomes. Recently, this susceptibility was linked to pH-dependent expression of the permease required for flucytosine uptake in A. fumigatus (11). Our data indicate that CO_2_ concentrations affect 5-FC activity toward A. fumigatus but that this effect indirect through modulation of medium pH. In contrast, we also found that the activity of flucytosine toward C. neoformans was increased by host-relevant concentrations of CO_2_ but was independent of pH. Finally, compared to standard *in vitro* susceptibility testing conditions, the expression of the Fcy2 permease required to import 5-FC appears to be expressed at much higher levels during mammalian infection, possibly explaining its significant *in vivo* efficacy compared to standard *in vitro* susceptibility testing Taken together, these observations suggest that antifungal testing under conditions that better approximate the host environment may be useful for some antifungal drugs, such as flucytosine.
